# Shock Wave Therapy Alleviates Hypoxia/Reoxygenation-Induced Cardiomyocyte Injury by Inhibiting Both Apoptosis and Ferroptosis

**DOI:** 10.1155/2024/8753898

**Published:** 2024-08-14

**Authors:** Jiannan Wang, Na Jia, Kaiyi Zhu, Kun Xu, Mingjing Yan, Ming Lan, Junmeng Liu, Bing Liu, Tao Shen, Qing He

**Affiliations:** ^1^ Department of Cardiology Beijing Hospital National Center of Gerontology Institute of Geriatric Medicine Chinese Academy of Medical Sciences, Beijing 100730, China; ^2^ Graduate School of Peking Union Medical College, Beijing, China; ^3^ Department of Cardiology Beijing Anzhen Hospital Capital Medical University, Beijing 100029, China; ^4^ Department of Cardiology Shanxi Bethune Hospital Shanxi Academy of Medical Sciences Tongji Shanxi Hospital Third Hospital of Shanxi Medical University, Taiyuan 030032, China; ^5^ The Key Laboratory of Geriatrics Beijing Institute of Geriatrics Institute of Geriatric Medicine Chinese Academy of Medical Sciences Beijing Hospital/National Center of Gerontology of National Health Commission, Beijing 100730, China

## Abstract

Shock wave therapy (SWT) is a new alternative therapy for patients with severe coronary artery disease that improves myocardial ischemic symptoms by delivering low-energy shock wave stimulation to ischaemic myocardium with low-energy pulsed waves. However, the specific mechanism of its protective effect is not fully understood, especially for the protective mechanism in cardiomyocytes after hypoxia/reoxygenation (H/R). We selected a rat H9c2 cardiomyocyte cell line to establish a stable H/R cardiomyocyte injury model by hypoxia/reoxygenation, and then used SWT for therapeutic intervention to explore its cardiomyocyte protective mechanisms. The results showed that SWT significantly increased cell viability and GSH levels while decreasing LDH levels, ROS levels, and MDA levels. SWT also improved mitochondrial morphology and function of cells after H/R. Meanwhile, we found that SWT could increase the expression of GPX4, xCT, and Bcl-2, while decreasing the expression of Bax and cleaved caspase-3, and inhibiting cardiomyocyte apoptosis and ferroptosis. Moreover, this protective effect of SWT on cardiomyocytes could be significantly reversed by knockdown of xCT, a key regulator protein of ferroptosis. In conclusion, our study shows that SWT can attenuate hypoxia–reoxygenation-induced myocardial injury and protect cardiomyocyte function by inhibiting H/R-induced apoptosis and ferroptosis, and this therapy may have important applications in the treatment of clinical myocardial ischemic diseases.

## 1. Introduction

The incidence of cardiovascular disease (CVD) continues to rise due to an aging population and has become both a leading cause of death and disability and a major socioeconomic burden on the healthcare system [1, 2]. Acute myocardial infarction (AMI) is one of the most rapidly progressive and lethal types of CVD. In its early stages, cardiomyocytes are damaged or even die due to ischemia and hypoxia. The main therapeutic options for ameliorating AMI include drug therapy, percutaneous coronary intervention, and coronary artery bypass grafting. These interventions can rapidly restore circulation to the ischemic myocardium, limit the size of myocardial infarction, and prevent the development of heart failure [3]. However, restoration of coronary blood flow in ischemic heart disease may lead to extra myocardial dysfunction, structural damage, and electrical disturbances, resulting in a significant increase in mortality. This phenomenon is called myocardial ischemia/reperfusion (I/R) injury [4], and it reduces the therapeutic advantage of the aforementioned interventions. Therefore, it is important to explore other treatment options for these patients.

Shock wave therapy (SWT) was first used as lithotripsy to treat kidney stones and gallstones in the 1980s [5, 6]. Subsequent studies found that high-frequency, low-energy SWT promoted neovascularization and accelerated tissue repair. Thus, the technique was gradually used in other fields such as orthopedics, plastic surgery, and rehabilitation medicine [7, 8, 9]. Cardiac shock wave therapy (CSWT), which was first developed in Switzerland in 2003, induces a series of biological effects by releasing shock waves into the ischemic area, generating cavitation, and mechanical shearing force [10]. Several studies have revealed that SWT could increase nitric oxide (NO) synthesis and endothelial nitric oxide synthase (eNOs) activity, upregulate the expression of vascular endothelial growth factor (VEGF), and enhance coronary angiogenesis in cell culture and animal models of acute or chronic myocardial ischemia [11, 12, 13]. Meanwhile, a growing number of clinical trials have demonstrated that CSWT could improve angina symptoms, stimulate neovascularization, improve exercise tolerance, and increase myocardial perfusion in patients with refractory angina and ischemic cardiomyopathy, suggesting that it is a promising technique for clinical application [14, 15, 16, 17].

Prior investigations have revealed that specific small molecules, including erastin and Ras-selective lethal 3 (RSL3), possess the capability to induce cell death via mechanisms divergent from apoptosis, and they are responsive to inhibition by iron chelators and antioxidants [18, 19]. In 2012, after thorough exploration and discussion of this alternative cell death pathway, Dixion et al. [20] introduced ferroptosis, a nonapoptotic cell demise mechanism reliant on iron. The morphological hallmarks of ferroptosis include reduced mitochondrial volume, increased density of double-layer membranes, and a decrease or absence of cristae. Molecular biological processes mainly manifest in the depletion of glutathione (GSH), decrease in the activity of glutathione peroxidase 4 (GPX4), production of a large number of reactive oxygen species (ROS), and accumulation of lipid peroxides in cells [21]. There is now growing evidence that ferroptosis plays a very important role in hypoxia/reoxygenation (H/R)-induced injury and that the obstruction of ferroptosis can lead to substantial protection of cardiomyocytes [22]. However, the clinical application of ferroptosis in the field of CVDs is still in its infancy, and as our understanding of the role of ferroptosis gradually deepens, this target may become the key to controlling the progression of H/R-related injury in the future [23].

Previous studies of our group and other groups have shown that SWT can protect cardiomyocytes from H/R-induced injury by inhibiting cell apoptosis [24, 25]. To the best of our knowledge, the relationship between ferroptosis and CSWT has not been reported in the literature. Thus, we hypothesize that SWT mitigates H/R-induced ferroptosis and apoptosis in H9c2 cardiomyocytes by upregulating the xCT/GPX4 pathway, enhancing intracellular GSH levels, reducing oxidative stress, and improving cell viability.

## 2. Materials and Methods

### 2.1. H9c2 Cell Culture

The H9c2 cardiomyocyte cell line used in this study was derived from a subclonal cell line of embryonic BD1X rat heart tissue from the American Type Culture Collection (ATCC, USA). H9c2 cells were cultured in complete Dulbecco's modified Eagle's medium (DMEM; Hyclone, USA), containing 10% (v/v) fetal bovine serum (FBS; Thermo Scientific, USA), 100 U/mL penicillin, and 100 *μ*g/mL streptomycin (Solarbio, China) under an atmosphere of 5% CO_2_ and 95% air (v/v) at 37°C. H9c2 cells were passaged when the cell confluence reached ~80%–95% (1 : 3 to 1 : 4). H9c2 cells were frozen in liquid nitrogen in 10% DMSO, 40% FBS, and 50% DMEM. For resuscitation, the cell freezing medium was rapidly thawed in water at 37°C. After centrifugation at 800 g for 6 min, cells were resuspended and cultured in a complete medium. All cell culture apparatuses were purchased from Corning.

### 2.2. H/R Model Establishment

The H9c2 cells were cultured in normoxia in 25-cm culture flasks. When the cell confluence reached 70%–80%, it was replaced with FBS-free RPMI1640 medium and cultured in a normoxia chamber for 24 hr to inhibit cell proliferation (synchronization). RPMI1640 medium was replaced with an appropriate amount of hypoxia-simulated buffer (in mM: 125 NaCl, 8 KCl, 1.2 KH_2_PO_4_, 1.25 MgSO_4_, 1.2 CaCl_2_, 6.25 NaHCO_3_, 5 sodium lactate, and 20 HEPES; pH 6.6). Cells were placed in a hypoxic chamber (Puhe Biotech, China), saturated with 94% N_2_, 5% CO_2_, and 1% O_2_ (v/v/v) at 37°C for 3, 5, 7, and 9 hr. After hypoxia, the medium was replaced with high-glucose DMEM medium containing 1% FBS, and the cells were cultured in a chamber containing 95% air and 5% CO_2_, modified from a previously reported study [26].

### 2.3. Shock Wave Treatment

After a certain period of incubation in hypoxic conditions, the posthypoxic H9c2 cell culture flasks were filled with serum-free medium. Then, the culture flasks were submerged in a 37°C water bath. The shock waves were generated from the CSWT system (Modulith SLC, Storz Medical, Switzerland) and focused on the cells through the water bath to avoid energy decay. The shock wave energy that we used to treat the cells was 0.07 mJ/mm^2^, the frequency was 3 Hz, and 200× SWT was given for each sample. After SWT, H9c2 cells were returned to the incubator with 95% air and 5% CO_2_ atmosphere for 12 hr at 37°C, modified from previously reported study [27].

### 2.4. Cell Viability Assay

3-(4,5-dimethylthiazole−2-yl)-2,5-diphenyltetrazolium bromide (MTT) was used to check cell viability under the guidance of the operating protocol (Beyotime, China). H9c2 cells were seeded in 24-well plates with or without SWT followed by H/R. After adding 5 mg/mL of MTT to each well, the cells were incubated in normoxia for 4 hr. Subsequently, the optical density (OD) was measured at 490 nm [26].

### 2.5. Cell Cytotoxicity Assay

Lactate dehydrogenase (LDH) is a terminal oxidoreductase of the glycolytic pathway, widely present in living mammalian cells, and can be released into the culture medium when cells are injured. Cell culture supernatants were harvested after H/R. The operation was performed according to the manufacturer's protocol of the LDH cytotoxicity assay kit (Solarbio, China). The OD was measured at 450 nm.

### 2.6. ROS Detection

H9c2 cells were stained with 10 *μ*M of dihydroethidium (DHE; Sigma–Aldrich, USA) for 30 min in a dark incubator at 37°C. ROS production was detected by fluorescence microscopy. The fluorescence intensity was proportional to the ROS level. Images were quantified with ImageJ software (NIH) [26].

### 2.7. GSH Content Detection

GSH can react with 5,5′-dithiobis-2-nitrobenoic acid to generate 2-nitro-5-mercaptobenzoic acid and oxidized glutathione glycide (GSSG). H9c2 cells harvested after H/R were operated according to the instructions of the GSH content assay kit (Solarbio), and the OD was measured at 412 nm.

### 2.8. Malondialdehyde (MDA) Content Detection

By evaluating the content of MDA, the level of lipid oxidation could be detected. The cells harvested after H/R were subjected to sample determination according to the protocol of the MDA content kit (Solarbio), and the OD was measured at 450 nm [28].

### 2.9. Western Blot Analysis

H9c2 cells were washed twice with ice-cold PBS and lysed by adding 1× cell lysis buffer (Cell Signaling Technology, CST, USA) including phosphatase and protease inhibitors (Solarbio). The protein concentration of the supernatant was determined with Pierce BCA Protein Kit (Thermo Fisher, USA). In total, 10–20 *μ*g of soluble proteins were separated by SDS–polyacrylamide gels (12%) and electrotransferred onto polyvinylidene fluoride (PVDF) membranes (Millipore, USA). Then, an appropriate amount of 5% skim milk prepared with TBS-T was added for 2 hr. The membranes were incubated with 1 : 1,000 dilution of primary antibodies overnight at 4°C. Primary antibodies used in the study included the following: anti-GAPDH (internal reference, Solarbio), anti-GPX4 (Abcam, UK), anti-xCT (Abcam), anti-Bcl-2 (Proteintech, China), anti-BAX (Proteintech), and anti-cleaved caspase-3 (Proteintech). The corresponding secondary antibody (1 : 5,000 dilution; ZSGB-BIO, China) was added for 1 hr at room temperature before ECL (Millipore) color development. Semiquantitative analysis of protein expression was performed using ImageJ software [28].

### 2.10. Cell Transfection

H9c2 cells were subjected to xCT siRNA (10 *μ*M; Genepharma, China) with GP-transfect–mate transfection reagent (Genepharma) according to the manufacturer's instructions [28]. First, xCT siRNA was diluted with serum-free medium (50 nM). Then, the transfection mixture was added to the H9c2 dish, and the cells were cultured at 37°C for 6 hr before being replaced with low-serum medium. After 24 hr of culture, cells were treated with hypoxia and SWT.

### 2.11. Transmission Electron Microscopy

The collected cells were fixed in phosphate-buffered glutaraldehyde (2.5%) and osmium tetroxide (1%), sectioned by an ultrathin microtome with a thickness of 50–70 nm, and stained with uranium acetate and lead citrate. Finally, observation and photographing were performed using a transmission electron microscope (Kylin-Bell Lab Instruments, Qilin, China) [29].

### 2.12. Statistical Analysis

The data are expressed as means ± standard errors (means ± SEMs). Statistical analyses were performed using SPSS 23.0 (IBM Corp., Chicago, IL, USA). Paired *t*-test was used for comparison between two groups, and one-way ANOVA was used for comparisons of multiple groups. Differences were considered statistically significant at *P*  < 0.05.

## 3. Results

### 3.1. H/R Decreases Cell Viability, Causes Cytotoxicity, and Promotes Both Apoptosis and Ferroptosis of H9c2 Cardiomyocytes

According to our team's previous findings, under the same hypoxic environment, H/R-induced autophagy peaked at 4–6 hr of hypoxia, and H/R-induced necroptosis was significantly elevated after 4 hr of hypoxia. We prove that SWT exerts a protective effect on H/R injury after incubation under hypoxia conditions for 5 hr [27, 30]. Therefore, we chose 3, 5, 7, and 9 hr of hypoxia as the initial exploratory experimental conditions.

**Figure 1:**
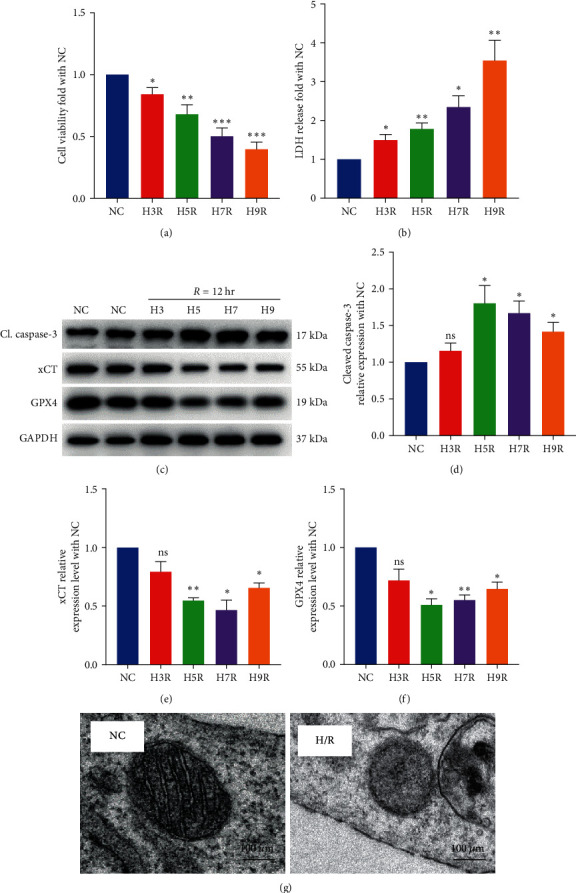
H/R decreased cell viability and promoted ferroptosis in H9c2 cardiomyocytes. (a) H/R reduced viability of H9c2 cardiomyocytes. The average MTT OD was measured at 490 nm (*n* = 7). (b) H/R promoted injury in H9c2 cardiomyocytes. The average LDH OD was measured at 450 nm (*n* = 5). (c–f) Western blot showing the expression levels of cleaved caspase-3, GPX4, and xCT in H9c2 cardiomyocytes after 0, 3, 5, 7, and 9 hr of hypoxia and 12 hr of reoxygenation (*n* = 3). (g) Transmission electron microscopy of mitochondria in H9c2 cardiomyocytes. The scales in the photographs represent 100 *μ*m (*n* = 3). Results are expressed as the mean ± SEM.  ^*∗*^*P* < 0.05,  ^*∗∗*^*P* < 0.01,  ^*∗∗∗*^*P* < 0.001 vs. NC.

The MTT method was used to measure the viability of H9c2 cells cultured with hypoxia-simulated buffer for 3, 5, 7, and 9 hr in a hypoxic incubator. The cell viability decreased with the increase in hypoxia time (Figure 1(a)), which was statistically significant compared with the normal control (NC) group. At 3, 5, 7, and 9 hr of hypoxia, the cell viability decreased by 14.9%, 31.8%, 49.4%, and 60%, respectively. We used the method of detecting the release of LDH to assess the cytotoxicity of H9c2 cells. The results showed that the degree of cytotoxicity increased with the prolongation of hypoxia time (Figure 1(b)), which was statistically significant compared with that in the NC group. The levels of LDH leakage in hypoxia conditions were 1.48, 1.79, 2.34, and 3.54 times higher than those in the NC group, respectively.

GPX4 is a selenoprotein that inhibits ferroptosis mainly by converting free H_2_O_2_ to H_2_O or reducing lipid hydrogen peroxide to lipid hydroxyl derivatives and converting GSH to GSSG; further, GPX4 is a hallmark regulator of ferroptosis [22]. The cystine/glutamate transporter (System Xc-) on the cell membrane contains two subunits, SLC3A2 and SLC7A11 (xCT), which maintain redox homeostasis by transferring cystine into the cell and glutamate out of the cell. The entry of cystine into the cytosol is degraded to cysteine for the synthesis of the antioxidant GSH, indicating that xCT is a negative regulator of ferroptosis [31]. In this study, the Western blot analysis was used to detect the expressions of GPX4, xCT, and cleaved caspase-3 with extended hypoxia time. The results showed that compared with the NC group, the levels of GPX4 and xCT began to decrease and the expression of cleaved caspase-3 began to increase significantly after 5 hr of hypoxia and 12 hr of reoxygenation (Figures 1(c), 1(d), 1(e), and 1(f)).

Observation of mitochondrial morphological changes under a transmission electron microscope is considered the “gold standard” to confirm the occurrence of ferroptosis [20]. In this study, compared with the NC group, transmission electron microscopy revealed that the H/R group had smaller mitochondria, decreased mitochondrial cristae, and increased density of the bilayer membrane, indicating that H9c2 cells underwent ferroptosis (Figure 1(g)).

### 3.2. SWT Increases Cell Viability and Inhibits Apoptosis Caused by H/R-Induced Injury in H9c2 Cardiomyocytes

**Figure 2:**
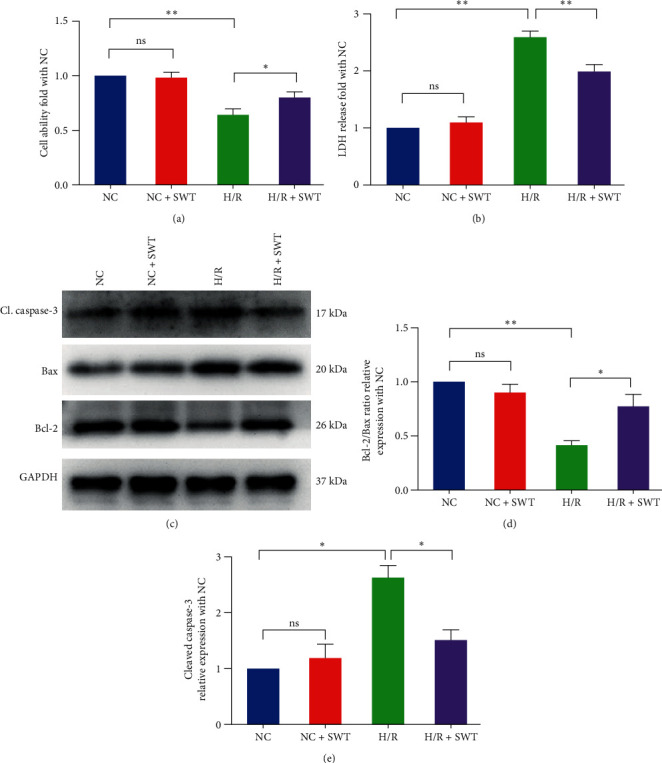
SWT increased cell viability and inhibited apoptosis induced by H/R injury in H9c2 cardiomyocytes. (a) SWT enhanced the viability of H9c2 cardiomyocytes during H/R. The average MTT OD was measured at 490 nm (*n* = 5). (b) SWT reduced injury of H9c2 cells during H/R. The average LDH OD was measured at 450 nm (*n* = 8). (c–e) SWT inhibited the expression level of cleaved caspase-3 and Bax, and it upregulated the expression level of Bcl2 (*n* = 3). Results are expressed as the mean ± SEM.  ^*∗*^*P* < 0.05,  ^*∗∗*^*P* < 0.01 vs. NC.

H9c2 cells were randomly divided into the NC group, NC + SWT group, H/R group, and H/R+SWT group. The MTT assay was used to measure the viability of H9c2 cells. The results showed that, compared with the H/R group, the average cell viability in the H/R + SWT group increased by 14.5% (*P*=0.025), and there was no significant change in the cell viability between the NC and NC + SWT groups (Figure 2(a)). The degree of cell cytotoxicity was measured by LDH release. The results showed that, compared with the H/R group, the average level of LDH release in the H/R + SWT group decreased by about 28.8% (*P*=0.004); there were no significant changes in LDH levels (Figure 2(b)). Based on our data, SWT increased cell viability and attenuated the cytotoxicity of H9c2 cardiomyocytes exposed to H/R.

The caspase family plays an essential role in the apoptotic process, with cleaved caspase-3 (the active form of caspase-3) being a key component of the final pathway leading to the onset of apoptosis [32]. Bax activates caspase-3 during apoptosis and promotes the process of apoptosis, while Bcl-2 inhibits this process and plays an antiapoptotic role [32]. The ratio of Bcl-2/Bax in H/R-induced H9c2 cells decreased (*P*=0.005), which was significantly inhibited by SWT (*P*=0.046). Compared with the NC group, the expression of cleaved caspase-3 in H/R-induced H9c2 cells significantly increased (*P*=0.017), while SWT could reduce the expression of cleaved caspase-3 in H/R-induced H9c2 cells (*P*=0.048) (Figures 2(c), 2(d), and 2(e)). Therefore, SWT played an antiapoptotic role in H/R-induced H9c2 cells.

### 3.3. SWT Decreases H/R-Induced Oxidative Stress

**Figure 3:**
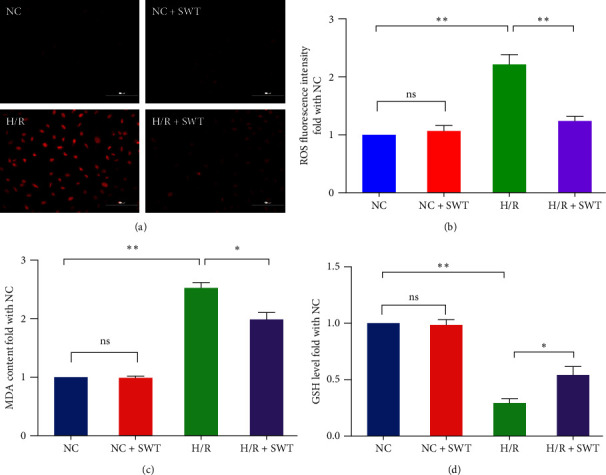
SWT decreased ROS production, lipid peroxidation, and oxidative stress induced by H/R injury in H9c2 cardiomyocytes. (a, b) SWT attenuated H/R-induced ROS levels in H9c2 cardiomyocytes (*n* = 4). (c) SWT decreased H/R-induced MDA levels in H9c2 cells. The average MDA OD was measured at 530 nm (*n* = 3). (d) SWT inhibited H/R-induced ferroptosis in H9c2 cardiomyocytes by increasing GSH (*n* = 3). Results are expressed as the mean ± SEM.  ^*∗*^*P* < 0.05,  ^*∗∗*^*P* < 0.01 vs. NC.

A large amount of ROS is generated during H/R, and Fe^2+^ converts H_2_O_2_ into hydroxyl radicals through the Fenton reaction, which further promotes the accumulation of ROS in cardiomyocytes. Fluorescence microscopy showed that the level of ROS in the H/R group was significantly higher than that in the NC group (*P*=0.006), and the ROS level in the H/R + SWT group was significantly lower than that in the H/R group (*P*=0.005). The NC + SWT group was similar to the NC group, and the ratios were not significantly different (Figures 3(a) and 3(b)). The level of lipid peroxidation in cardiomyocytes was detected by MDA content. The results showed that there was no significant change in the level of MDA in the NC + SWT group, compared with the NC group. The MDA level in the H/R group was 2.52 times higher than that in the NC group (*P*=0.004). Compared with the H/R group, the MDA level in the SWT group decreased by 21% (H/R+SWT group vs. H/R group: 1.99 ± 0.21 vs. 2.52 ± 0.16, *P*=0.034; Figure 3(c)). Therefore, H/R-mediated ROS and MDA production were inhibited by SWT.

GSH is an important antioxidant in the human body and a cofactor of GPX4, and it maintains the balance of free radicals in cells. Thus, GSH can reduce harmful lipid hydroperoxides, produced by cell metabolism, to a harmless lipid alcohol, scavenging free radicals, and thereby inhibiting the occurrence of ferroptosis. Therefore, inactivation of GSH/GPX4 increases intracellular lipid peroxidation and induces ferroptosis. In the study, by measuring the GSH content in H9c2 cells, we showed that the GSH content in the H/R group was significantly lower than that in the NC group (*P*=0.003). Compared with its effect in the H/R group, SWT could significantly increase the GSH content in H9c2 cells induced by H/R by about 1.85 times (*P*=0.042), thereby reducing the occurrence of ferroptosis (Figure 3(d)).

### 3.4. SWT Alleviates H/R-Induced Ferroptosis in H9c2 Cardiomyocytes

**Figure 4:**
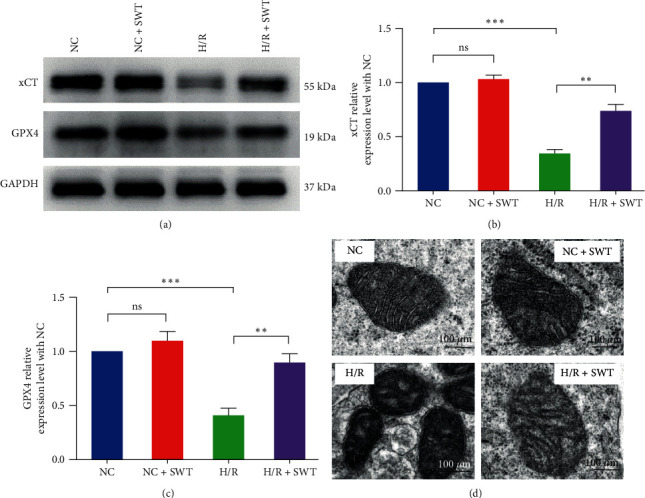
SWT alleviated H/R-induced ferroptosis in H9c2 cardiomyocytes. (a–c) SWT alleviated H/R-induced ferroptosis in H9c2 cardiomyocytes via the xCT/GPX4 signaling pathway. Expression levels of GPX4 and xCT were shown by Western blot in the NC, NC + SWT, H/R, and HR + SWT groups (*n* = 3). (d) Effect of SWT on mitochondrial morphology of H9c2 cardiomyocytes by transmission electron microscopy in the NC, NC + SWT, H/R, and H/R + SWT groups. The scales in the photographs represent 100 *μ*m (*n* = 3). Results are expressed as the mean ± SEM.  ^*∗∗*^*P* < 0.01,  ^*∗∗∗*^*P* < 0.001 vs. NC.

Compared with the H/R group, the expression levels of xCT and GPX4 in H9c2 cells were significantly increased after SWT (*P*  < 0.01), while there was no significant change between those in the NC and NC + SW groups (Figures 4(a), 4(b), and 4(c)). Compared with the NC and NC + SWT groups, the mitochondrial volume of the cardiomyocytes in the H/R group was reduced, the mitochondrial cristae were reduced, and the double-layer membrane density was increased. After SWT, the mitochondrial morphology was improved, and the mitochondria were larger and had more mitochondrial cristae (Figure 4(d)).

### 3.5. Knockdown of xCT Reverses the Inhibitory Effect of SWT on Ferroptosis in Cardiomyocytes

**Figure 5:**
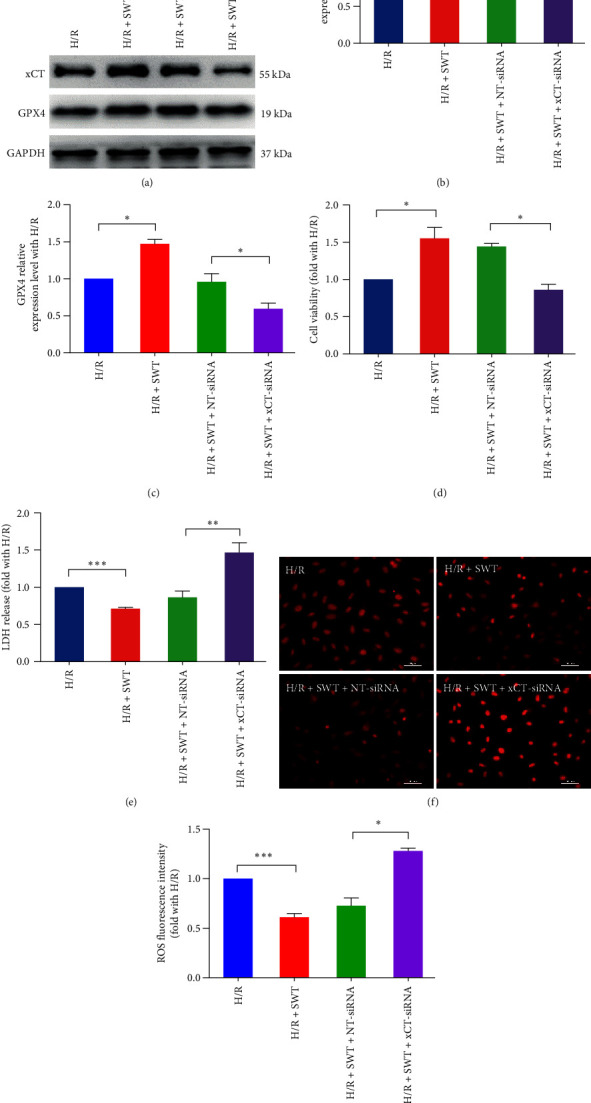
Knockdown of xCT expression reversed the inhibitory effect of SWT on ferroptosis in H9c2 cardiomyocytes. (a–c) Western blot and average expression levels of xCT and GPX4 in the H/R, H/R + SWT, H/R + SWT + NT-siRNA, and H/R + SWT + xCT-siRNA groups (*n* = 3). (d, e) The cell viability of cardiomyocytes, as detected by MTT assay (*n* = 3), and cell cytotoxicity, as evaluated by LDH method (*n* = 5), in the H/R, H/R + SWT, H/R + SWT + NT-siRNA, and H/R + SWT + xCT-siRNA groups. (f, g) DHE staining for H9c2 cells in the H/R, H/R + SWT, H/R + SWT + NT-siRNA, and H/R + SWT + xCT-siRNA groups (*n* = 3). Results are expressed as the mean ± SEM.  ^*∗*^*P* < 0.05,  ^*∗∗*^*P* < 0.01,  ^*∗∗∗*^*P* < 0.001 vs. NC.

To investigate whether ferroptosis is an important treatment target of SWT, we transfected xCT siRNA to reduce the expression of xCT. The expressions of xCT and GPX4 were detected by Western blot. The results showed that the expressions of xCT and GPX4 in the H/R + SWT group were significantly higher than those in the H/R group (*P*  < 0.05). After knockdown of xCT, SWT could not promote the expression of xCT and GPX4, and the difference was statistically significant compared with the H/R + SWT and the H/R + SWT + NT-siRNA group (NT stands for nontargeting siRNA, *P*  < 0.05; Figures 5(a), 5(b), and 5(c)). Compared with that in the H/R group, the cell viability of the H/R + SWT + xCT-siRNA group was slightly decreased, the leakage of LDH was increased, and the level of ROS was increased (Figures 5(d), 5(e), and 5(f)). Therefore, knockdown of xCT could reverse the protective effect of SWT on H/R-induced ferroptosis, which suggests that xCT and ferroptosis may play an important role in the cardiomyocyte protective function of SWT.

## 4. Discussion

To the best of our knowledge, this is the first report to assess the effect of SWT on ferroptosis in cardiomyocytes exposed to H/R. In the present study, we found that by establishing the H/R model of H9c2 cells, H/R could increase cell injury and induce ferroptosis and apoptosis. SWT alleviated H/R-induced cell injury, increased cell viability, reduced oxidative stress and lipid peroxidation levels, and inhibited both ferroptosis and apoptosis after H/R.

In acute myocardial infarction (AMI), H/R-related damage, an inevitable risk factor, is closely associated with ferroptosis [33]. Importantly, several studies have demonstrated that inhibition of ferroptosis alleviates cardiac reperfusion injury [34, 35, 36, 37, 38]. The major process of ferroptosis involves the massive accumulation of iron-dependent ROS, which exceeds the cell's ability to maintain redox homeostasis, leading to lipid peroxidation and ultimately cell death. ROS and lipid peroxidation in lipid metabolism play important roles in H/R-induced ferroptosis and are associated with a variety of programed cell deaths [39]. Previous studies have shown that SWT can inhibit cardiomyocyte apoptosis and necroptosis by reducing ROS [24, 30]. The present study showed that SWT had a similar effect, which could inhibit H/R-induced ferroptosis by reducing the levels of ROS and lipid peroxidation.

Extracellular iron can enter cardiomyocytes through multiple pathways, and cardiomyocytes are susceptible to iron accumulation under pathological conditions. Fe^2+^ in the unstable iron pool acts as a catalyst in ferroptosis by inducing ROS production, thereby making cardiomyocytes more sensitive to oxidative stress in the presence of iron overload [21]. The xCT/GPX4 pathway is the main regulatory mode of ferroptosis. On the cell membrane, System Xc- constitutes a heterodimer consisting of SLC7A11 (xCT) and SLC3A2, with primary functionality attributed to xCT. It facilitates the exchange of extracellular cystine with intracellular glutamate at a 1 : 1 ratio, thus furnishing the essential substrate for intracellular glutathione (GSH) synthesis [40]. GPX4, an enzyme reliant on GSH, converts it to GSSG, effectively eliminating excessive peroxides and hydroxyl radicals produced during cellular respiration and metabolism. Consequently, GPX4 holds a crucial position in suppressing lipid peroxidation. Hindrance to GPX4′s activity gives rise to ROS formation and lipid peroxidation, eventually triggering ferroptosis [22]. Dixion et al. [20] transfected siRNA to decrease the expression of xCT, resulting in a substantial increase in sensitivity to erastin-induced ferroptosis in HT-1080 cells. Conversely, overexpression of xCT in HT-1080 cells significantly blocked the occurrence of ferroptosis. Jiang et al. [41] noted that the activation of p53 led to a decline in GPX4 activity, hampered cystine uptake, suppressed xCT expression, heightened ROS production, and ultimately prompted ferroptosis. Yu et al. [42] established a rat myocardial I/R injury model and administered dexmedetomidine (DEX) at the beginning of reperfusion to alleviate I/R-induced myocardial injury, reduce ROS levels, and alleviate mitochondrial dysfunction. The activation of the xCT/GPX4 pathway inhibited ferroptosis, and erastin reversed this protective effect. Xu et al. [43] found that H/R-induced ferroptosis in rat myocardium and H9c2 cells and naringenin could protect cardiomyocytes through the Nrf2/xCT/GPX4 pathway, while erastin reversed their protective effect. Thus, inhibition of the Nrf2/xCT/GPX4 pathway reduced intracellular GSH levels, resulting in decreased GPX4 activity and ultimately ferroptosis. Similarly, our study found that H/R could reduce the expression of xCT/GPX4 and GSH content in H9c2 cells, which could be markedly ameliorated by SWT. SiRNA-mediated knockdown of xCT enhanced ferroptosis during H/R injury in H9c2 cells and reversed this protective effect by SWT. Therefore, SWT inhibited H/R-induced ferroptosis through xCT/GPX4 pathway.

SWT not only inhibited H/R-induced apoptosis but also ferroptosis. Several studies have shown that apoptosis can be converted into ferroptosis under certain conditions and that ferroptosis can promote cellular sensitivity to apoptosis by mechanisms that may be related to p53 and endoplasmic reticulum stress [44, 45, 46]. p53 is the “guardian of the genome” and regulates ferroptosis through transcriptional or posttranslational mechanisms in addition to affecting apoptosis, autophagy, and the cell cycle. On the one hand, p53 promotes ferroptosis by repressing xCT expression and upregulating spermidine/spermine N1-acetyltransferase (SAT1) and glutaminase 2 (GLS2) expression; on the other hand, p53 inhibits ferroptosis by directly repressing dipeptidyl peptidase 4 (DPP4) activity or inducing CDKN1A/p21 expression [41, 47]. In addition, Li et al. [48] found that propofol inhibited ferroptosis via the AKT/p53 signal pathway in an I/R model in vivo by knocking down AKT. Our findings suggest that SWT inhibited the expression of proapoptotic proteins BAX and cleaved caspase-3, and it promoted the expression of antiapoptotic protein Bcl2 to regulate apoptosis, which is consistent with previous studies [24, 49]. However, the mechanism of how SWT affects the relationship between apoptosis and ferroptosis needs further exploration.

Due to its nonrenewable nature, maintenance of the number of cardiomyocytes is essential to maintain the structural integrity and function of the heart. Myocardial ischemia and hypoxia will lead to a loss of cardiomyocytes and decreased cardiac function. Histology methods have shown that inhibition of cardiomyocyte apoptosis and/or ferroptosis reduces myocardial infarct size and improves postischemic cardiac insufficiency in I/R injury models, which also provides a new cardioprotective target for patients with AMI [35, 38, 50]. Based on the results of this study, SWT may be one of the alternative therapies to conventional myocardial injury treatment and has important applications for the treatment of clinical myocardial ischemic diseases.

## 5. Conclusions

In conclusion, SWT can inhibit both apoptosis and ferroptosis in H/R-induced cardiomyocyte injury model by alleviating lipid peroxidation and promoting xCT/GPX4 signaling pathways. SWT may be an effective alternative treatment for preventing I/R injury in patients with AMI.

## Data Availability

The datasets used and/or analyzed during the current study are available from the corresponding author upon reasonable request.
